# Clinical aspects of reimbursement policies for orphan drugs in Central and Eastern European countries

**DOI:** 10.3389/fphar.2024.1369178

**Published:** 2024-03-08

**Authors:** Szczepan Jakubowski, Pawel Kawalec, Przemyslaw Holko, Iwona Kowalska-Bobko, Maria Kamusheva, Guenka Petrova, Pero Draganić, Leos Fuksa, Agnes Männik, Fanni Ispán, Vitalis Briedis, Ioana Bianchi, Marian Sorin Paveliu, Tomas Tesar

**Affiliations:** ^1^ Department of Health Promotion and e-Health, Faculty of Health of Science, Jagiellonian University Medical College, Kraków, Poland; ^2^ Department of Nutrition and Drug Research, Faculty of Health of Science, Jagiellonian University Medical College, Kraków, Poland; ^3^ Health Policy and Management Department, Faculty of Health of Science, Jagiellonian University Medical College, Kraków, Poland; ^4^ Department of Organization and Economics of Pharmacy, Faculty of Pharmacy, Medical University of Sofia, Sofia, Bulgaria; ^5^ Agency for Medicinal Products and Medical Devices of Croatia, Zagreb, Croatia; ^6^ Department of Biotechnology, University of Rijeka, Rijeka, Croatia; ^7^ Department of Social and Clinical Pharmacy, Faculty of Pharmacy in Hradec Kralove, Charles University in Prague, Prague, Czechia; ^8^ Institute of Family Medicine and Public Health, University of Tartu, Tartu, Estonia; ^9^ Department of Reimbursement, National Institute of Health Insurance Fund Management, Budapest, Hungary; ^10^ Department of Clinical Pharmacy of Lithuanian, University of Health Sciences, Kaunas, Lithuania; ^11^ The Romanian Association of International Medicine Manufacturers. ARPIM, Bucharest, Romania; ^12^ Faculty of Medicine, Titu Maiorescu University, Bucharest, Romania; ^13^ Department of Organisation and Management in Pharmacy, Faculty of Pharmacy, Comenius University in Bratislava, Bratislava, Slovakia

**Keywords:** orphan drugs, rare diseases, clinical, policy, reimbursement, HTA, CEE

## Abstract

**Objectives:** The aim of this study was to characterize the reimbursement policy for orphan drugs (ODs) in Central and Eastern European (CEE) countries in relation to the availability and impact of clinical evidence, health technology assessment (HTA) procedure, selected economic indicators, and the drug type according to indications.

**Materials and methods:** A list of authorized medicines with orphan designation and information about active substance, Anatomical Therapeutic Chemical (ATC) classification, and therapeutic area was extracted from the web-based register of the European Medicines Agency (EMA). A country-based questionnaire survey was performed between September 2021 and January 2022 in a group of selected experts from nine CEE countries (an invitation was sent to 11 countries). A descriptive and statistical analysis was conducted to determine statistical significance, correlations, between the drug or country characteristic and the positive recommendation or reimbursement of ODs**.**

**Results:** The proportion of reimbursed orphan drugs differed between countries, ranging from 17.7% in Estonia to 49.6% in Hungary (*p* < 0.001). The odds that ODs were reimbursed were reduced in countries with a “strong” level of impact of drug safety and efficacy on reimbursement decisions (*p*=0.018), the presence of other additional specific clinical aspects (e.g., genomic data) considered in the reimbursement decision (*p* < 0.001) and mandatory (without exception) safety assessments (*p*=0.004). The probability that ODs were reimbursed was increased in countries with a “moderate” level of impact of drug safety and efficacy on reimbursement decisions (*p*=0.018), when reimbursement decisions are dependent on the EMA registration status and orphan drug designation (*p* < 0.001), the presence of the “positive HTA recommendation guarantees reimbursement” policy (*p* < 0.001), higher GDP per inhabitant (*p*=0.003), and higher healthcare expenditure (*p* < 0.001).

**Conclusion:** We found that there are differences among CEE countries in the reimbursement of orphan drugs, and we identified aspects that may influence these differences. Safety, efficacy, and specific clinical aspect issues significantly influenced reimbursement decisions. Antineoplastic and immunomodulating agents drugs were the largest group of ODs and increased the chance of getting a positive recommendation. The higher GDP per inhabitant and healthcare expenditures per inhabitant were positively linked to the chance that an OD receives reimbursement.

## 1 Introduction

Orphan drugs (ODs) are medications used to treat rare diseases. In the European Union (EU), rare diseases are defined as chronic or life-threatening conditions with a prevalence of less than 5 in 10,000 people (Winstone et al., 2015; European Medicines Agency, 2023). The essential formal requirements for developing orphan drugs to benefit patients and facilitate new therapies, along with incentives for the pharmaceutical industry, are included in the European Parliament and Council Regulation No. 141/2000 (European Parliament and Council, 2000; Michel and Toumi, 2012).

In Europe, there are not many medicines with an orphan designation. In 2017, there were 95 ODs (Malinowski et al., 2018), and the number had increased to 133 by 2021 (Orphanet, 2021). Between 2017 and 2021, the European Medicines Agency (EMA) authorized 72 new drugs with an orphan designation: 13 drugs in 2017, 21 in 2018, 6 in 2019, 20 in 2020, and 12 in 2021 (Orphanet, 2021; Orphanet, 2022). Orphan designation has been granted for 10 years by the Committee for Medicinal Products for Human Use (CHMP) of the EMA (European Parliament and Council, 2000). Therefore, there are more drugs for rare diseases, but not all of them have an orphan designation—the designation may have expired or the manufacturer may not have applied for it.

Surprisingly, the type of disease treated with ODs can affect the drug policy. Malinowski et al. (2018) showed that a country’s drug policy (e.g., shares of reimbursed ODs and funding decisions) may be different depending on drug indication. For example, in Germany, 68% of ODs for metabolic diseases were reimbursed, as compared with 97% of ODs for oncologic diseases (*p* < 0.01). Moreover, the 12 European countries included in the study differed in policy instruments for ODs in terms of pricing and managed entry agreements. For example, there were significant differences observed in the shared OD reimbursement, with the lowest share in Poland (27%) and the highest share in Denmark (88%) (*p* < 0.0001) (Malinowski et al., 2018).

Differences in orphan drug policies are also caused by the fact that many governments developed their own legislation and strategies to support and encourage the research and development of new drugs (Garau, 2009; Blankart et al., 2011). Regarding pricing and reimbursement, factors with the greatest influence on decision-makers include disease severity and the lack of an alternative treatment option. The most important concerns, on the other hand, are the cost of therapy and limited evidence base (Garau, 2009). As for insufficient scientific evidence, Pontes et al. (2018) identified major uncertainties regarding the assessment of clinical trials on orphan drugs. These include in some cases:

– lack of clinical trials (data come from bibliographic reports, observational retrospective studies, or compassionate programs instead);
– in the absence of the required data, the use of results from negative trials only, which consider a therapeutic or other intervention ineffective or presentation of low-quality evidence in primary studies;
– use of intermediate variables as the main endpoint;
–drawing conclusions on the basis of incomplete or *ad hoc* analyses;
– evaluation of clinical safety in an insufficiently sized group.

Although the overall trend has been decreasing, there are still some differences in the drug policy between Western European and Central and Eastern European (CEE) countries, mainly in terms of pharmaceutical expenditure and budget (Jakovljevic et al., 2016). As for the CEE countries, their drug policies are generally similar, with some minor differences. According to Kawalec et al. (2017), similarities can be found in mechanisms, such as positive reimbursement lists, external and internal reference pricing, risk-sharing schemes, and an obligatory health technology assessment (HTA) dossier, for submitting a pricing and reimbursement application. There are also similarities in an orphan drug policy where CEE countries implement special reimbursement regulations, which, in turn, affect HTA agency’s decision-making processes and HTA requirements (Malinowski et al., 2018).

Although HTA plays an important role on reimbursement decisions in many countries, it does not guarantee the availability of orphan drugs for patients (Zamora et al., 2019); in some cases, positive HTA recommendations do not necessarily lead to faster access to new drugs. The process may be delayed by a prior budget impact analysis of medicines, price negotiations, and the time required for the recommendation to be implemented by local commissioning authorities (Zamora et al., 2019).

In our study, cross-country comparisons were used to characterize the reimbursement policy in CEE countries in relation to the availability and impact of clinical evidence, HTA procedure, selected economic indicators, and Anatomical Therapeutic Chemical (ATC) classification. We considered the differences and similarities in drug policies according to the reimbursement statuses/recommendations of all ODs to identify factors responsible for access to ODs for patients. In addition, the aim of this study was to show the differences in CEE countries from the economic perspective (gross domestic product [GDP], healthcare expenditures, and pharmaceutical spending) and assess their influence on the reimbursement of ODs.

## 2 Materials and methods

In the initial phase of the study, a list of authorized medicines with orphan designation and information about active substance, ATC classification (WHO, 2022), and the therapeutic area was obtained from the EMA’s web-based register (as of September 2021) (European Medicines Agency, 2022). Country-level information, such as the GDP, percentage of GDP spending on pharmaceuticals, and healthcare expenditures across CEE countries, was collected from the Eurostat and the Organization for Economic Cooperation and Development (OECD) (Eurostat, 2022; OECD, 2022).

A questionnaire survey was conducted among reimbursement/drug market access experts from selected CEE countries to obtain detailed information on the implications of reimbursement policies for ODs. The survey was carried out from September 2021 to January 2022, and the invitation was sent to a group of 24 experts from 11 countries. Only experienced experts were invited to participate; they were identified as the co-authors of scientific publications on OD reimbursement and market access in MEDLINE and Google Scholar. Next, the following criteria were considered while the experts selected such as a degree in science or academia and demonstrated expertise with market access or reimbursement of ODs and practical experience in related fields. Finally, selected experts were accepted only if they declared no conflict of interest within the scheduled study.

The questionnaire contained open and closed questions. A three-level scale was used to answer questions regarding impact (low, moderate, and strong). The scale was not quantitatively or qualitatively defined, and the expert’s task was to subjectively assess which answer was the most applicable, according to his or her knowledge. A scope of questions followed the research objective. The questionnaire assessed the following aspects related to orphan drugs:

a) HTA recommendations and reimbursement statuses;
b) clinical aspects (safety and efficacy) considered in the reimbursement decision-making;
c) drug policy mechanisms and strategies;
d) HTA dossier use in the reimbursement procedure.

The qualitative analysis of collected data resulted in the descriptive country profiles of the reimbursement policy and HTA procedure for ODs. Data on the number of ODs with positive recommendations and reimbursed status in each country were descriptively analyzed and presented as frequencies or percentages. The analyses were performed separately for the subgroups of conditions identified by ATC classification. The χ^2^ Pearson test was used to compare the OD status between countries (i.e., OD reimbursed or not; with or without a positive recommendation for reimbursement). A series of logistic regression models with nested random effects (drug variable within the country variable) and a single fixed effect were performed to identify the characteristics of the countries and drugs that may be associated with the reimbursement status or a positive recommendation for the reimbursement of ODs across CEE countries. The selection and assessment of the mixed-effect models were based on the log-likelihood function. Models with nested random effects were implemented due to the design of the study (the same set of ODs evaluated within each country) and the attempt to identify aspects that are most likely correlated with the reimbursement status or positive recommendation across CEE countries but not limited to the ODs that are tested. All models had a significant, non-zero variance between countries and a non-zero variance between ODs, suggesting the occurrence of other aspects, not measured in this study, that are correlated with the positive recommendation or reimbursement status of ODs. Multivariate models were not tested because the characteristics of countries and ODs were often correlated and/or dependent on each other. Only positive recommendations were considered because, in some countries, information on drug evaluation with a negative recommendation is not published. Concordance between the positive recommendation and drug reimbursement status was assessed by a percentage agreement and Cohen’s κ-coefficient. A *p*-value of less than 0.05 was considered significant. Data were prepared and analyzed using Stata 17SE (StataCorp., College Station, TX, United States) and OriginPro 2021b (OriginLab Corporation, Northampton, MA, United States).

## 3 Results

As of September 2021, there were 125 drugs with orphan designation on the EMA’s web-based register (Supplementary Appendix S1). According to the ATC classification, orphan drugs were classified (listed in the order of the largest group) as follows:

–ATC L (“antineoplastic and immunomodulating agents,” n = 44);
– ATC A (“alimentary tract and metabolism,” n = 23);
– ATC B (“blood and blood-forming organs,” n = 10);
– ATC J (“anti-infectives for systemic use,” n = 10);
– ATC N (“nervous system,” n = 10);
– ATC M (“musculoskeletal system,” n = 5);
–ATC H (“systemic hormonal preparations, excluding sex hormones and insulins,” n = 4);
– ATC R (“respiratory system,” n = 4);
– ATC S (“sensory organs,” n = 4);
– ATC C (“cardiovascular system,” n = 3);
– ATC D (“dermatologicals,” n = 2);
– ATC V (“various,” n = 2).

Four drugs (n = 4) were not assigned to any of the ATC classifications by EMA, and we were unable to find information on this in any other sources. In statistical analysis, the ATC group which has less than or equal to five orphan drugs (ATCs: M, H, R, S, C, D, and V) was combined into a single group named “other ATCs.” Economic and demographic information at the national level in selected CEE countries is presented in Table 1.

**TABLE 1:** Selected economic characteristics for the surveyed CEE countries.

Characteristic	Bulgaria	Croatia	Czechia	Estonia	Hungary	Lithuania	Poland	Romania	Slovakia
Population, 2021^a^	6,916,548	4,036,355	10,701,777	1,330,068	9,730,772	2,795,680	37,840,001	19,201,662	5,459,781
Healthcare expenditure and PPS, per inhabitant, 2019^a^	1,316.56	1,439.61	2,442.58	1,791.88	1,550.80	1,949.20	1,636.24	1,354.42	1,564.59
GDP and PPS, per inhabitant, 2020^a^	16,400	19,200	27,800	25,200	22,100	26,000	22,600	21,500	20,900
Pharmaceutical spending, total, and % of GDP, 2019^b^	2.45	1.41	1.19	1.12	1.73	1.57	1.27	1.48	1.70

Source:

^a^ Eurostat 2021.

^b^ OECD 2021

GDP, gross domestic product; PPS, purchasing power standard.

Completed survey questionnaires were received from 11 respondents (this represents 46% of all experts invited) from nine countries (no data gathered from Latvia & Slovenia). Completed questionnaires were obtained from the following countries: Bulgaria (number of included experts; n = 2), Croatia (n = 1), Czechia (n = 1), Estonia (n = 1), Hungary (n = 1), Lithuania (n = 1), Poland (n = 1), Romania (n = 2), and Slovakia (n = 1). The background of the experts was as follows: eight people represented academia, one person represented both university and a national drug evaluation agency, one person was affiliated with a national health insurance institute, and one expert with an international association of drug manufacturers.

### 3.1 Recommendations and reimbursements of ODs in CEE countries

The comparison of the number of ODs with a positive recommendation and the number of those drugs being reimbursed in each CEE country is presented in Table 2. An estimate of 23.8% of orphan drugs had positive recommendation to be reimbursed across countries studied (*p* < 0.001). Romania had the highest number of ODs with a positive recommendation (44.0%), while Estonia had the lowest (8.0%; *p* < 0.001) across all studied countries. Orphan drugs obtained from the ATC L category (“antineoplastic and immunomodulating agents”) received the highest number of positive recommendations across CEE countries (35.2%; *p* < 0.001) compared to all ATC categories. Across all studied countries, Hungary had the highest number of reimbursed orphan drugs (49.6%), followed by Czechia (48.8%; *p* < 0.001)—but in both countries, ODs assessed individually outside the standard reimbursement procedure were also included. On the other hand, Estonia had only 17.7% of ODs reimbursed across all studied countries (*p* < 0.001)—including hospital procedures with orphan drug administration—and if only out-patient medicines are considered, it will be equal to 8.0%. Overall, 32.3% of ODs were reimbursed across countries studied (*p* < 0.001). Orphan drugs from ATC L (“antineoplastic and immunomodulating agents”) received the highest number of reimbursements across CEE countries (42.3%; *p* = 0.011).

**TABLE 2:** Number of orphan drugs (share of all included) having a positive recommendation and those being reimbursed by country and ATC classification.

	Anatomical therapeutic chemical	Bulgaria	Croatia	Czechia	Estonia	Hungary	Poland	Romania	Slovakia	% in all countries	*p*-value
**Positive recommendation**	ATC A	4 (16.7%)	8 (33.3%)	5 (21.7%)	0 (0.0%)	0 (0.0%)	6 (25.0%)	8 (33.3%)	2 (8.3%)	(17.3%)	**0.003**
	ATC B	2 (20.0%)	2 (20.0%)	2 (22.2%)	0 (0.0%)	2 (20.0%)	0 (0.0%)	4 (40.0%)	0 (0.0%)	(15.2%)	0.137
	ATC J	3 (30.0%)	5 (50.0%)	2 (20.0%)	2 (20.0%)	1 (10.0%)	1 (10.0%)	3 (30.0%)	2 (20.0%)	(23.8%)	0.476
	ATC L	15 (34.1%)	20 (45.5%)	18 (43.9%)	5 (11.4%)	15 (34.1%)	16 (36.4%)	28 (63.6%)	6 (13.6%)	(35.2%)	**<0.001**
	ATC N	2 (20.0%)	1 (10.0%)	3 (33.3%)	1 (10.0%)	0 (0.0%)	1 (10.0%)	4 (40.0%)	0 (0.0%)	(15.2%)	0.120
	other ATCs^a^	3 (11.5%)	11 (40.7%)	3 (11.5%)	2 (7.4%)	3 (11.1%)	5 (18.5%)	8 (29.6%)	2 (7.4%)	(17.3%)	**0.009**
	Total	29 (23.4%)	47 (37.6%)	33 (28.0%)	10 (8.0%)	21 (16.8%)	29 (23.2%)	55 (44.0%)	12 (9.6%)	(23.8%)	**<0.001**
**Reimbursement**	ATC A	4 (16.7%)	8 (33.3%)	10 (41.7%)	2 (8.3%)	11 (45.8%)	3 (12.5%)	6 (25.0%)	6 (25.0%)	(26.0%)	**0.026**
	ATC B	2 (20.0%)	2 (20.0%)	4 (40.0%)	2 (20.0%)	3 (30.0%)	3 (30.0%)	1 (11.1%)	4 (40.0%)	(26.6%)	0.810
	ATC J	3 (30.0%)	5 (50.0%)	4 (40.0%)	2 (20.0%)	5 (50.0%)	0 (0.0%)	3 (30.0%)	2 (20.0%)	(30.0%)	0.217
	ATC L	15 (34.1%)	20 (45.5%)	26 (59.1%)	12 (27.9%)	25 (56.8%)	16 (36.4%)	21 (48.8%)	13 (29.6%)	(42.3%)	**0.011**
	ATC N	2 (20.0%)	1 (10.0%)	5 (50.0%)	1 (10.0%)	5 (50.0%)	0 (0.0%)	3 (30.0%)	0 (0.0%)	(21.3%)	**0.016**
	other ATCs^a^	3 (11.5%)	11 (40.7%)	12 (44.4%)	3 (11.1%)	13 (48.2%)	5 (18.5%)	6 (22.2%)	9 (33.3%)	(28.8%)	**0.005**
	Total	29 (23.4%)	47 (37.6%)	61 (48.8%)	22 (17.7%)	62 (49.6%)	27 (21.6%)	40 (32.5%)	34 (27.2%)	(32.3%)	**<0.001**

Bold values mean than p-values are less than 0.05

^a^ Other ATCs: M, H, R, S, C, D, and V

Note: Data on reimbursed ODs, and ODs with positive recommendations from Lithuania were not obtained. Frequencies are shown in brackets.

There was a substantial agreement between positive recommendation and reimbursement status for all orphan drugs across CEE countries (Cohen’s κ of 0.644, *p* < 0.001). The percentage of agreement ranged from 64% in Hungary (κ of 0.276, *p* < 0.001) to 89.4% in Romania (κ of 0.778, *p* < 0.001) and 100% in Bulgaria and Croatia (Supplementary Appendix S2).

### 3.2 Clinical aspects in the reimbursement decision-making, reimbursement policy, and HTA procedure for orphan drugs in CEE countries

In addition to the results presented in Tables 3, 4, below are detailed the country-specific characteristics of the clinical aspects in reimbursement decision-making, the reimbursement policy, and HTA procedure for ODs.

**TABLE 3:** Safety, efficacy, and other aspects of orphan drugs in the reimbursement policy in selected CEE countries.

Characteristic	Bulgaria	Croatia	Czechia	Estonia	Hungary	Lithuania	Poland	Romania	Slovakia
Safety assessment (not required, partially, and mandatory)	Mandatory	Mandatory	Mandatory	Mandatory	Partially	Partially	Mandatory	Partially	Partially
Definition of the acceptable safety profile	No	Yes	No	No	No	No	No	No	No
Reimbursement without sufficient evidence of safety	No	No	No	No	No	No	No	No	Yes
Similar safety profile to pediatric and adult assessment	Yes	Yes	Yes	Yes	No	Yes	Yes	Yes	Yes
Efficacy assessment (not required, partially, and mandatory)	Mandatory	Mandatory	Mandatory	Mandatory	Mandatory	Mandatory	Mandatory	Partially	Partially
Definition of the acceptable efficacy profile	No	No	No	No	No	Yes	No	No	No
Reimbursement without sufficient evidence of efficacy	Yes	Yes	Yes	No	No	No	No	No	Yes
Similar efficacy profile to pediatric and adult assessment	Yes	Yes	Yes	Yes	Yes	Yes	Yes	No	Yes
Impact of safety and efficacy on reimbursement decisions (low, moderate, and strong)	Strong	Strong	Moderate	Strong	Moderate	Strong	Strong	Low	Moderate
Other clinical aspects influencing the reimbursement process	Yes	No	No	No	No	Yes	Yes	No	Yes
Dedicated to OD legislation and policies	Yes	No	Yes	No	No	Yes	No	Yes	Yes
Reimbursement decisions dependent on EMA registration status and OD designation	No	No	No	No	Yes	Yes	No	Yes	No

EMA, European Medicines Agency; ODs, orphan drugs.

**TABLE 4:** HTA procedure and analyses for orphan drugs in selected CEE countries.

Characteristic	Bulgaria	Croatia	Czechia	Estonia	Hungary	Lithuania	Poland	Romania	Slovakia
Health Technology Assessment (HTA) (not required, partially, and mandatory)	Mandatory	Partially	Mandatory	Mandatory	Mandatory	Mandatory	Mandatory	Partially^c^	Partially
Institution applying for reimbursement	MAH or authorized representative	MAH	MAH	MAH, doctors, and organizations	MAH and manufacturers	MAH, hospital, or similar specialized care	MAH	MAH	MAH
Special advisory institution/-s	Yes	No	Yes	No	Yes	Yes	Yes	Yes	Yes
Positive HTA recommendation guarantees reimbursement	No	Yes	Yes^a^	Yes^a^	Yes^a^	No	No^b^	Yes	No
ICER/ICUR thresholds the same for orphan drugs as for non-orphan drugs	Yes	n/a	Yes	Yes	No	Yes	Yes	n/a	No
					Higher threshold for RD therapies				Higher threshold for ODs
Budget impact analysis	+	+	+	+	+	+	+	-	+
Clinical analysis	+	+	+	-	+	+	+	-	+^d^
Cost-benefit analysis	+	-	-	-	-	-	-	-	-
Cost-effectiveness analysis	+	+	+	+	+	+	+	-	+^d^
Cost-minimization analysis	+	-	-	+	-	-	+	-	+^d^
Cost-utility analysis	+	-	+	+	+	+	+	-	+^d^

ICER, incremental cost-effectiveness ratio; ICUR, incremental cost utility ratio; MAH, marketing authorization holder; n/a, not applicable.

^a^ Some ODs obtained a reimbursement without positive HTA recommendations.

^b^ Some ODs obtained a reimbursement with negative HTA recommendations.

^c^ “HTA-like,” based on reports elaborated in three countries (France, Denmark, and the United Kingdom).

^d^ “partially” because there is no need to submit information about relative efficacy, cost-effectiveness, etc., for the treatment of a disease whose prevalence in Slovakia is less than 1:50,000.

^e^ The rules apply precisely for the treatment of rare diseases (RDs), where ODs (drugs with EMA’s orphan designation) are included but non-orphan drugs may also appear.

In Bulgaria, all ODs had to show a general acceptable safety profile to be reimbursed, while there were some exceptions for the efficacy profile (such as when drugs were on the “positive drug list” with an obligation to monitor the effect of therapy). One of the requirements for reimbursement was a recommendation from the National Council on Prices and Reimbursement (the advisory institution) that is positive. A managed entry agreement with the public payer was a second requirement. Bulgaria had some special legislation and policies about orphan drugs regarding specific HTA criteria (e.g., additional evidence of the benefits from administering the medicinal product, Markov modeling, assessment based on the severity of the rare condition, and existence of an alternative). Clinical aspects affecting Bulgarian reimbursement decisions included OD subgroups or categories identified based on genomic data and information on the moral and ethical aspects of health technologies used. In addition to HTA, analyses and modeling based on the systematic reviews/meta-analysis of published clinical trials were being performed.

In Croatia, the definition of an acceptable safety profile for an orphan drug was used and could be translated as the benefits of drug application outweigh the risk of adverse effects. There was no definition of an acceptable efficacy profile because it depends on the opinion of the experts and is applied case-by-case. All ODs had to show a generally acceptable safety profile to be reimbursed, while there were some exceptions for the efficacy profile (in individual cases for specific diseases). ICER/ICUR thresholds were not used for OD reimbursement decisions because of a limited number of patients and the lack of clinical evidence. Although the HTA assessment was not obligatory for reimbursement decision, it was applied in many cases.

In Czechia, safety and efficacy evaluations were mandatory, but their impact on reimbursement decisions was assessed as “moderate,” and this is because the evaluation was done on the same basis as for non-orphan drugs. There was no formal safety evaluation procedure dedicated to ODs if a drug was authorized for marketing in EU, and efficacy data were generally considered sufficient. Reimbursement was never refused solely because of insufficient efficacy, unless there was a non-matching population suggested for reimbursement (i.e., a different population for which the efficacy data were lacking). These data may be supplemented in the reimbursement process with observational data and/or case reports. Czechia had some special legislation and policies regarding ODs: medicines may be granted 2 + 1 year of temporary reimbursement without mandatorily meeting the general single cost-effectiveness threshold (i.e., 47,000 EUR per QALY); the orphan-specific path into reimbursement with a decision-making body (namely, without cost-effectiveness driving the decision and also broader “societal” criteria to be considered, e.g., social security benefits and employment). Reimbursement decisions for some ODs, especially those for ultra-rare diseases, may bypass the general reimbursement system (guarantees of positive recommendation) by implementation to extraordinary ways of public coverage. However, those drugs were practically reimbursed but on an individual case-by-case scheme. Additionally, this individual reimbursement (of a generally and visibly “non-reimbursed” drug) was in the remit of National Insurance Funds, who decide individually. Medical scientific societies have acted as advisors, but from January 2022, a new advisory body was established (the State Institute for Drug Control) for OD reimbursement in Czechia and the reimbursement process focused more on clinical data analysis.

In Estonia, beside the standard reimbursement path being very specific, orphan drugs could be provided to patients (and indirectly “reimbursed”) also via hospital procedures where the costs of drugs were included. Such ODs did not have to go through the HTA procedure, and no recommendation in such cases was provided. For other scenarios, positive recommendations based on HTA guaranteed OD reimbursement for out-patient medicines (ambulatory drugs).

In Hungary, the safety assessment was included in the reimbursement process, but no additional evaluation criteria (compared to non-orphans) were considered. There were differences in safety profile assessments between ODs for children and adults: for pediatric ODs, the profile was evaluated on a case-by-case basis. On the other hand, there were no such differences for the efficacy profile assessment. However, apart from the standard reimbursement procedure, patients could get access to ODs (with a high level of reimbursement and minimal patient co-payment) within the framework of the Named Patient Program (NPP). NPP was outside the framework of the regular reimbursement (no HTA dossier required), dedicated to unique patient needs and medical conditions. The level of reimbursement/co-payment was based on the National Institute of Health Insurance Fund Management (NIHIFM) decisions in every case, which on an average was 95%–98%—NIHIFM can also provide drug recommendations for reimbursement. The reimbursement was accepted individually for every patient request, permissions were valid for 3–12 months, and before the end of the validity, patients have to request for extension. There was a possibility for the request rejection and NPP did not mean automatic access to OD therapies. A positive recommendation from an HTA agency influenced reimbursement decisions; however, it did not provide a full guarantee because it depends on the available budget. In Hungary, a higher ICER threshold was used for ODs compared with non-orphan drugs. These thresholds applied to the treatment of rare diseases, where ODs (drugs with EMA’s orphan designation) were included but non-orphan drugs could also be included. In addition, safety analysis and value-added analysis were conducted for reimbursement decisions, and HTA was sometimes affected by conditional reimbursement.

In Lithuania, no specific rules for ODs were applied for the safety assessment in the reimbursement process as EU marketing authorization was considered sufficient for such an assessment. Only EMA-approved drugs were considered candidates for reimbursement. Lithuania had used a definition of an acceptable efficacy profile of an OD, which could be translated as a drug that effectively prolongs survival or reduces disability by addressing the etiological and pathogenetic factors. Lithuania had special legislation and policies regarding ODs; special policies were approved by the order of the Minister of Health, and rules applied for including orphan drugs into the list of medicines used for rare disease therapy. Reimbursement advice was provided by a special reimbursement commission. There were some additional clinical aspects affecting reimbursement evaluation, such as survival and/or reduction of patient disability by effectively addressing the causative factors, including etiological factors (i.e., those that determine the onset of the disease) and/or pathogenetic factors (i.e., those that determine the clinical course of the disease). Other important aspects, such as a therapeutic benefit or compliance of the medicinal product with the orphan medicine status were also emphasized. Additional information was used on the compliance of the medicinal product with OD status and the therapeutic benefit delivered by the medicinal product, based on the data provided from clinical trials and other relevant medical information.

Poland did not have special legislation or policies regarding orphan drugs, but at the time of survey, we revealed the draft national rare disease plan (the goal of the plan is to improve diagnostics, increase access to drugs, and launch patient registries). The advisory institution on drug reimbursement in Poland is the national HTA agency (Agency Of Medical Technology Assessment And Tariff System). There were some additional clinical aspects affecting reimbursement evaluations, such as a quality of life, overall survival and safety in relation to current standard therapy (comparator), innovation of the drug, and lack of an alternative treatment method (breakthrough therapy). In Poland, additional analyses called “rationalization analyses” have to be provided to the Ministry of Health in case a budget impact analysis revealed an increase in the reimbursement costs due to the positive reimbursement of the analyzed therapeutic methods. An objective of that is to provide the Ministry of Health with suggestions on changes in the reimbursement system in Poland by providing savings which would balance the additional budget on the new drug reimbursement (ISAP, 2023). Decision problem analysis and preferably cost-utility analysis with the assessment for cost/QALY were used in HTA on a regular basis.

In Romania, the HTA analysis was not conducted by national institutions but was based on the HTA dossier from other countries. There were no differences in the safety profile assessments between ODs for children and adults, but in the case of efficacy, drugs were evaluated differently, considering the risks and capabilities of young patients (e.g., some drugs were reimbursed only for adults). Romania had some special policies regarding ODs, which were not in terms of legislation but there were special criteria for assessing ODs based on the orphan drug registration status and reimbursement in EU member states; cost comparison is not requested. In addition, there were specific criteria for evaluating new plasma-derived drugs for the treatment of rare diseases, for which there are no alternatives. Advisory institutions (an HTA department from the National Medicines Agency) played an important role in orphan drug reimbursement; an “HTA-like” dossier was required based on the number of points scored on specific criteria (HTA decisions from the United Kingdom, France, and Germany; the number of EU countries with reimbursement; the development of a local real-world data study; and a budget impact assessment). In OD assessment, a specific score card solution was also applied. This score card evaluated the annual cost of treatment (cost comparison vs. comparator) per patient.

In Slovakia, there were some exceptions where ODs were reimbursed despite not showing a general acceptable safety (considering the assessment of the safety as an issue for the marketing authorization procedure). Additionally, a significant number of ODs were included into the Slovak reimbursement list without pharmacoeconomic dossiers. There is no legislation, which can be used to refuse the reimbursement based on the safety in the case that orphan drugs received marketing authorization from EMA (Tesar et al., 2019). In Slovakia, a Reimbursement Committee was established as an advisory body supported by the working groups (e.g., medical experts and health-economic consultants). There were also some exceptions for ODs which were reimbursed in spite of an acceptable efficacy profile; such reimbursement is available due to legislation, indicating no need to submit information about relative efficacy and cost-effectiveness of drugs for diseases with a prevalence of less than 1:50,000 in a country. Slovakia had special policies regarding ODs: a “backdoor” market access for expensive drugs, a disease-specific scheme for cancer and orphan drugs, and no legal requirement to prove their cost-effectiveness. QALYs for a limited number of ODs (as additional clinical aspects) affected reimbursement evaluation. If a marketing holder submitted the dossier for reimbursement in Slovakia, the drug was automatically included into the reimbursement list without relevant discussion about efficacy, effectiveness, safety, or cost-effectiveness (without a HTA dossier) (Tesar et al., 2019). Budget impact analysis was fully used in the orphan drug assessment, while other HTA analyses had partial importance. In Slovakia, ODs used a higher ICER threshold compared to non-orphans. Additional (clinical) elements in HTA included relative effectiveness (i.e., QALY gain); disease severity; safety impact on the society if not treated (e.g., spread of infection); social value; whether it was a first or second option or adjunctive treatment; risk of abuse; whether it was a causal, prophylactic, or symptomatic treatment, which lastly impacts on the total costs.

### 3.3 Aspects associated with orphan drug reimbursement

Several drug and country characteristics were found to be associated with the probability that an orphan drug has a positive recommendation or is reimbursed across CEE countries (Table 5). ATC classification; the level of impact of the safety and efficacy assessment on reimbursement decisions; dedicated to OD legislations and policies; the presence of other clinical aspects influencing reimbursement; orphan registration or designation status from EMA; “positive HTA recommendation guarantees reimbursement” policy; and GDP per inhabitant was significantly correlated with the chance of positive recommendation. The level of impact of the safety and efficacy assessment on reimbursement decisions; the presence of safety assessment during the reimbursement process, the presence of other clinical aspects influencing reimbursement; orphan registration or designation status from EMA; “positive HTA recommendation guarantees reimbursement” policy; healthcare expenditure per inhabitant; and GDP per inhabitant were significantly correlated with the chance of reimbursement across CEE countries.

**TABLE 5:** Univariate associations between the drug or country characteristic and the positive recommendation or reimbursement of ODs.

Characteristic		Positive recommendation		Reimbursement	
		OR (95% CI)	*p*-value	OR (95% CI)	*p*-value
Anatomical Therapeutic Chemical (ATC) classification	[ATC B vs. ATC A]	0.94 (0.23–3.82)	0.932	1.17 (0.19–7.36)	0.869
	[ATC J vs. ATC A]	1.55 (0.39–6.09)	0.534	1.07 (0.17–6.83)	0.944
	[ATC L vs. ATC A]	3.56 (1.41–8.97)	**0.007**	3.41 (0.98–11.84)	0.054
	[ATC N vs. ATC A]	0.99 (0.25–3.99)	0.989	0.78 (0.12–4.94)	0.791
	[Other ATCs vs. ATC A]	1.12 (0.40–3.15)	0.837	1.17 (0.30–4.66)	0.819
	[Overall effect]	—	**0.034**	—	0.281
Impact of safety and efficacy assessment on reimbursement orphan drug (OD) decisions	[Moderate vs. low]	0.15 (0.09–0.27)	**<0.001**	2.05 (1.13–3.71)	**0.018**
	[Strong vs. low]	0.23 (0.14–0.40)	**<0.001**	0.49 (0.27–0.88)	**0.018**
	[Overall effect]	—	**<0.001**	—	**<0.001**
Dedicated to orphan drug legislation and policies	[Yes vs. no]	1.45 (1.02–2.05)	**0.036**	1.12 (0.79–1.59)	0.539
Other clinical aspects influencing orphan drug reimbursement	[Yes vs. no]	0.52 (0.36–0.75)	**0.001**	0.31 (0.21–0.47)	**<0.001**
Safety assessment	[Mandatory vs. partially]	1.04 (0.73–1.49)	0.825	0.58 (0.40–0.84)	**0.004**
Efficacy assessment	[Mandatory vs. partially]	0.84 (0.16–4.52)	0.836	1.21 (0.23–6.40)	0.816
Reimbursement decisions dependent on European Medicines Agency registration status and orphan drug designation	[Yes vs. no]	1.93 (1.31–2.84)	**0.001**	2.61 (1.73–3.94)	**<0.001**
Health technology assessment (HTA)	[Mandatory vs. partially]	0.46 (0.11–1.85)	0.273	0.88 (0.20–3.90)	0.867
Positive HTA recommendation guarantees reimbursement	[Yes vs. no]	1.93 (1.33–2.80)	**0.001**	3.18 (2.13–4.76)	**<0.001**
ICER/ICUR thresholds the same for ODs as for non-ODs	[Yes or n/a vs. no]	2.79 (0.77–10.08)	0.118	0.44 (0.08–2.53)	0.355
Healthcare expenditure per inhabitant	[Increase of 10^3^ PPS]	0.54 (0.33–1.01)	0.054	2.76 (1.64–4.65)	**<0.001**
Gross domestic product (GDP) per inhabitant	[Increase of 10^3^ PPS]	0.95 (0.90–1.00)	**0.048**	1.09 (1.03–1.15)	**0.003**
% of GDP spending on pharmaceuticals	[Increase of 1%]	0.90 (0.59–1.40)	0.648	0.81 (0.52–1.26)	0.346

Bold values mean than p-values are less than 0.05.

ICER, incremental cost-effectiveness ratio; ICUR, incremental cost utility ratio; CI, confidence interval; OR, odds ratio; PPS, purchasing power standard.

Note: All models had significant (*p* < 0.05), non-zero between-country variance and non-zero between-drug variance, which suggests the occurrence of other aspects, not measured in this study, that are correlated with positive recommendation or reimbursement of ODs.

**FIGURE 1:**
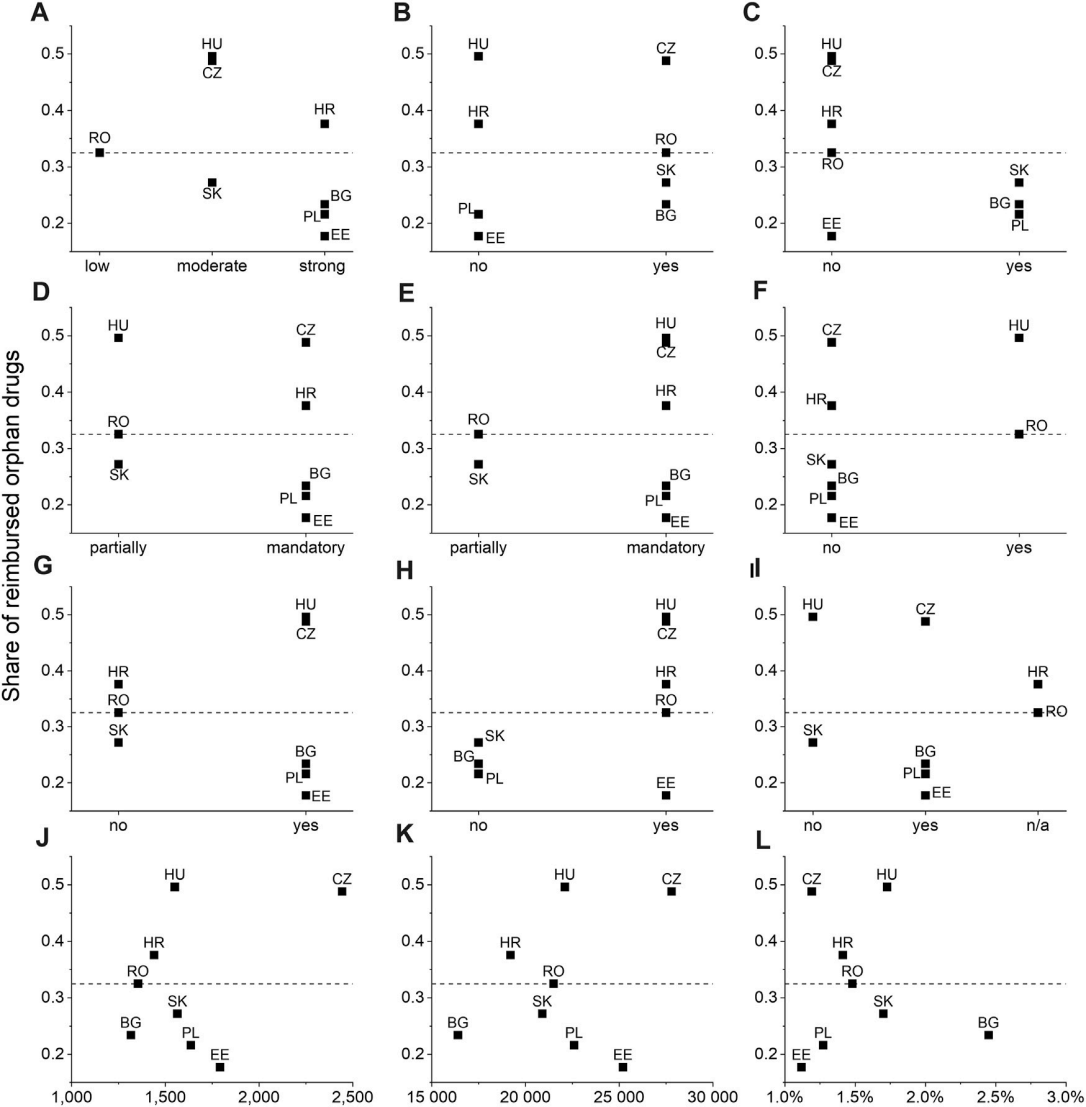
Share of ODs reimbursed in selected countries according to a country attribute, i.e., the impact of safety and efficacy assessment on reimbursement decisions **(A)**, the presence of dedication to OD legislation and policies **(B)**, the presence of other clinical aspects influencing reimbursement **(C)**, safety assessment during the reimbursement process **(D)**, efficacy assessment during the reimbursement process **(E)**, reimbursement decisions depend on the EMA registration status and OD designation **(F)**, formal requirement for the HTA dossier **(G)**, positive HTA recommendation guarantee reimbursement **(H)**, same ICER/ICUR threshold for ODs and non-ODs **(I)**, healthcare expenditure per inhabitant, in PPS, 2019 **(J)**, GDP per inhabitant, in PPS, 2020 **(K)**, and the share of GDP spent on pharmaceuticals, 2019 **(L)**. BG, Bulgaria; CZ, Czechia; EE, Estonia; HR, Croatia; HU, Hungary; PL, Poland; RO, Romania; SK, Slovakia. The average across all countries (0.323) is indicated by a dashed line. Data on reimbursed ODs from Lithuania were not obtained.

The share of OD reimbursed varied according to some of the country’s characteristics (drug policy and economic indicators) (Figure 1). In countries with the highest share of reimbursed orphan drugs, such as Czechia (includes ODs in non-standard reimbursement) and Hungary (includes ODs in NPP), the impact of safety and efficacy assessment was rated as “moderate,” while in countries with the lowest share of reimbursed orphan drugs, such as Estonia (includes healthcare procedure with ODs) and Poland, the impact was rated as “strong.” The results showed that countries both with a high and low share of reimbursed orphan drugs either had or did not have a special policy and legislation for ODs. In countries with the highest share of reimbursed ODs (Czechia and Hungary) and those with the lowest share of reimbursed ODs (Poland and Estonia), HTA dossiers were obligatory in the reimbursement process. Additionally, countries in which a positive recommendation ensured reimbursement (such as Hungary and Czechia) had a higher share of reimbursed orphan drugs, as compared with countries where a positive recommendation did not ensure reimbursement (such as Poland and Bulgaria).

## 4 Discussion

The main objective of this study was to characterize the reimbursement policy for ODs in CEE countries in relation to the availability of clinical evidence and the impact of the HTA procedure, selected economic indicators, and drug type (based on the indication). The similarities and differences on OD reimbursement across CEE countries were revealed. Both the number of orphan drugs with a positive recommendation to be included in the list of reimbursed drugs and the number of orphan drugs being reimbursed significantly differed between CEE countries. It appeared that the drugs policy that requires any level of efficacy or safety assessment of ODs was negatively correlated with the chances that the drug being recommended and, in some cases, even reimbursed. This may be due to the fact that ODs had usually more limited clinical evidence than other drugs (i.e., registration trials with a smaller population size and only surrogate endpoints) (Joppi et al., 2006; Dupont and Van Wilder, 2011), and the assessment of those drugs according to the standards established for other drugs may fail. A better understanding of the characteristic of the evidence for ODs, shown by a special policy of its evaluation, can result in a higher probability of a positive HTA recommendation. We observed that the higher GDP and the healthcare expenditure per inhabitant increased the probability of ODs, having positive recommendation or being reimbursed in CEE countries. In addition, certain groups of drugs had a higher probability to receive positive recommendation by up to three times (“antineoplastic and immunomodulating agents” vs. “alimentary tract and metabolism”).

We have identified some relevant publications for comparison with our findings. Malinowski et al. (2019) evaluated the OD reimbursement decision-making processes in selected CEE countries (n = 10). The proportion of reimbursed orphan drugs differed between countries, ranging from 6.3% to 27.4%—in our study, it was 17.7%–49.6%. This might be commented as an improvement in patients’ access to ODs. According to researchers, a full or condensed HTA of the submitted reimbursement application was required in most studied countries—similar to our findings. The authors found that half of the studied countries had specific rules in force for the reimbursement of ODs, and no government adopted a higher ICER criterion for ODs. In our study, dedicated policies for ODs were adopted by more than half countries, and in two countries (Hungary and Slovakia), the ICER threshold appeared to be higher for orphan drugs (treatment rare diseases) compared to standard drugs. Additionally, Malinowski et al. (2019) found out that the proportion of ODs that were reimbursed varied across countries (by the type of disease and treatment, budgetary resources), but no association with GDP was found. This contrasts with our study, which showed that an increase in GDP per each 1,000 PPF increased the chances of a reimbursement and that some groups of ODs for specific conditions (ATC classification) indeed received a reimbursement more frequently than others.

In a more recent study, Malinowski et al. (2020) assessed the proportion of HTA recommendations and reimbursement decisions for ODs used to treat oncologic diseases and provided a detailed description of country-specific HTA policies. The authors emphasized that, in general, ODs need to be cost-effective, have an acceptable safety profile, and have sufficient efficacy, but other factors were also considered when reaching the final decision on reimbursement—consistent with our observations. Our study differed where we analyzed ODs for all conditions, did not assess the impact of individual EMA registration statuses, and did not include negative recommendations for reimbursement in the analysis.

Our results are in line with the study proposed by Kamusheva et al. (2018), which showed that reimbursement requirements were similar in CEE countries, and there was a tendency for the systems to be developed in accordance with the most recent standards for scientific, pharmacoeconomic, and HTA requirements. The authors investigated the availability of biotechnological ODs, legislative pricing and reimbursement requirements, HTA, and reimbursement costs. Similar to our study, the researchers noted that reimbursement decisions were based on conventional requirements that were valid for all medicines (sometimes specific additional criteria were used). They also revealed that the final reimbursement decisions in some CEE countries were based on reports from other governments (Romania did so in our study), that the access has improved recently (study from 2018) in countries studied, and that the proportion of ODs that were reimbursed in a given country was linked to the time of accession to EU.

In a systematic review, Zelei et al. (2016) focused on the perspective of third-party payers in CEE countries to examine the scientific evidence on value drivers for the evaluation of orphan drugs as HTA in the reimbursement process. The authors developed a list of potential value drivers of ODs in CEE countries, including disease-related, treatment-related, economic, and social factors. Some of these factors were consistent with the clinical aspects that were assessed in our study (e.g., safety and efficacy), as well as with some HTA aspects (e.g., ICER) and other determinants (e.g., social impact and QALYs) identified by experts as having impact on recommendations or reimbursement decisions. The authors concluded that orphan drug pricing and reimbursement in the CEE region should be more open and supported by data. We agree with this, especially, when our study shows the magnitude of the impact of efficacy and safety assessments (based on available data) was linked to positive recommendations and reimbursement decisions. In addition, Zelei et al. (2016) proposed that legislative mechanisms should be established to mitigate the harmful effects of patient accessibility to ODs on external pharmaceutical price referencing systems.

Our study has several limitations. We assessed drugs with an orphan designation granted in 2021, and OD policies in CEE countries may have changed since then, along with dynamic changes in expenditure, drug policy strategies, and access to orphan drugs. In Hungary, for example, there have been numerous changes since 2021 in drug applications (new indications and new rules in reimbursement recommendations) in favor of an increased availability of ODs. We were unable to collect all relevant data for Lithuania because of restrictions on access to public data. In addition, due to different data collection methods and data format, information on OD spending in CEE countries could not be used and standardized. There are not many local (country) experts in the area of orphan drugs that are accessible and involved in research, and some of them may work in public and private sectors. Having confidence in the answers obtained from the experts, we cannot exclude some inaccuracies due to a subjective perspective. We cannot exclude the possibility that other factors may also have affected the observed results. Therefore, it is necessary to evaluate other aspects that may be equally important for the reimbursement of ODs, such as drug prices, manufacturer investment in a given country, or social and ethical factors. In some cases, the coefficient might have been affected by the levels and frequencies of the analyzed variables. Thus, it should be treated as a descriptive rather than an inferential statistic. Although we allowed the interception of models to be different for each country, the impact of, for example, a special reimbursement policy for ODs was not directly adjusted for the difference in, for example, the GDP per inhabitant. Multivariate models were not tested because the characteristics of the countries and drugs were often correlated with and/or dependent on each other.

Despite the above limitations, our study addresses an important issue, namely, the impact of clinical aspects within reimbursement policies on access to ODs. Another major strength of the study is that it shows the impact of the safety, efficacy, and other aspects of ODs on reimbursement decisions. Moreover, data are unique (sometimes difficult to access because of a language barrier) and reliable because they were collected in collaboration with experienced experts who are familiar with the country-specific issues of the reimbursement policy. Our study showed similarities and a few differences in the evaluation of clinical evidence (safety, efficacy, and other aspects) between CEE countries. These results remain valid because policy changes in Europe (joint clinical assessment for ODs) that could possibly affect the drug evaluation process for ODs are not expected until 2028 (EUR Lex, 2021). Nevertheless, further research could provide more data on some other clinical aspects of OD reimbursement policies in other countries.

## 5 Conclusion

Our study indicated differences between CEE countries in the reimbursement of orphan drugs, and we identified aspects that may influence these differences, as reflected by the number of drugs that received a positive recommendation and reimbursement. Safety, efficacy, and specific clinical aspect issues significantly influenced reimbursement decisions. Hence, not only a special reimbursement pathway for ODs, the level of impact of efficacy and safety evidence, and the presence of other clinical aspects but also the GDP and healthcare expenditures were significantly associated with the probability of a positive recommendation and a reimbursement decision in CEE countries. Our study revealed that Antineoplastic and immunomodulating agents drugs were the largest group of ODs and increased the chance of getting a positive recommendation. The higher GDP per inhabitant and healthcare expenditures per inhabitant were positively linked to the chance that an OD receives reimbursement.

## Data Availability

The raw data supporting the conclusions of this article will be made available by the authors, without undue reservation.
