# Supplementary material for: Clinical aspects of reimbursement policies for orphan drugs in Central and Eastern European countries
Source: Front Pharmacol. 2024 Mar 8;15:1369178. doi: 10.3389/fphar.2024.1369178 (PMC10957562; doi:10.3389/fphar.2024.1369178)
Supplement: Supplementary file 1 [file Table1.DOCX]

# Attachments

ONLINE ONLY SUPPLEMENTARY INFORMATION

# Appendix 1. | The EMA-registered orphan drugs (valid for September 2021) – alphabetical order.

|  | **Trade name** | **International non-proprietary name (INN) or common name** | **Active substance** | **Anatomical Therapeutic Chemical (ATC)** | **Therapeutic area (MeSH)** |
| --- | --- | --- | --- | --- | --- |
| 1 | Abecma | Idecabtagene vicleucel | idecabtagene vicleucel | L01 | Multiple Myeloma |
| 2 | Adakveo | Crizanlizumab | Crizanlizumab | B06AX01 | Anemia, Sickle Cell |
| 3 | Adcetris | Brentuximab vedotin | Brentuximab vedotin | L01XC12 | Lymphoma, Non-Hodgkin |
| 4 | Adempas | Riociguat | Riociguat | C02KX05 | Hodgkin Disease |
| 5 | Alofisel | Darvadstrocel | Darvadstrocel | L04 | Rectal Fistula in Crohn’s disease |
| 6 | Alprolix | Eftrenonacog alfa | Eftrenonacog alfa | B02BD04 | Hemophilia B |
| 7 | Amglidia | Glibenclamide | Glibenclamide | A10BB01 | Diabetes Mellitus |
| 8 | Arikayce liposomal | Amikacin | Amikacin sulfate | J01GB06 | Respiratory Tract Infections |
| 9 | Ayvakyt | Avapritinib | avapritinib | L01EX18 | Gastrointestinal Stromal Tumors |
| 10 | Besponsa | Inotuzumab ozogamicin | Inotuzumab ozogamicin | L01XC | Precursor Cell Lymphoblastic Leukemia-Lymphoma |
| 11 | Blenrep | Belantamab mafodotin | belantamab mafodotin | L01XC39 | Multiple Myeloma |
| 12 | Blincyto | Blinatumomab | Blinatumomab | L01XC | Precursor Cell Lymphoblastic Leukemia-Lymphoma |
| 13 | Brineura | Cerliponase alfa | Cerliponase alfa | A16AB | Neuronal Ceroid-Lipofuscinoses |
| 14 | Bronchitol | Mannitol | Mannitol | R05CB16 | Cystic Fibrosis |
| 15 | Bylvay | Odevixibat | Odevixibat | A05AX | Cholestasis, Intrahepatic |
| 16 | Cablivi | Caplacizumab | Caplacizumab | B01A | Purpura, Thrombotic Thrombocytopenic |
| 17 | Cerdelga | Eliglustat | Eliglustat | A16AX10 | Gaucher Disease |
| 18 | Chenodeoxycholic acid Leadiant | Chenodeoxycholic acid | Chenodeoxycholic acid | A05AA01 | Xanthomatosis, Cerebrotendinous; Metabolism, Inborn Errors |
| 19 | Coagadex | Human coagulation factor X | Human coagulation factor X | B02BD13 | Factor X Deficiency |
| 20 | Cometriq | Cabozantinib | Cabozantinib | L01XE | Thyroid Neoplasms |
| 21 | Cresemba | Isavuconazole | Isavuconazole | J02AC | Aspergillosis |
| 22 | Crysvita | Burosumab | Burosumab | M05BX05 | Hypophosphatemia, Familial Hypophosphatemic Rickets, X-Linked Dominan |
| 23 | Cystadrops | Mercaptamine | Mercaptamine hydrochloride | S01XA21 | Cystinosis |
| 24 | Dacogen | Decitabine | Decitabine | L01BC08 | Leukemia, Myeloid |
| 25 | Darzalex | Daratumumab | Daratumumab | L01XC24 | Multiple Myeloma |
| 26 | Daurismo | Glasdegib | Glasdegib maleate | L01XX63 | Leukemia, Myeloid, Acute |
| 27 | Defitelio | Defibrotide | Defibrotide | B01AX01 | Hepatic Veno-Occlusive Disease |
| 28 | Deltyba | Delamanid | Delamanid | J04AK06 | Tuberculosis, Multidrug-Resistant |
| 29 | Dovprela | Pretomanid | Pretomanid | J04 | Tuberculosis, Multidrug-Resistant |
| 30 | Elzonris | Tagraxofusp | tagraxofusp | L01XX67 | Lymphoma |
| 31 | Enspryng | Satralizumab | satralizumab | L04AC | Neuromyelitis Optica |
| 32 | Epidyolex | Cannabidiol | Cannabidiol | N03AX | Lennox Gastaut Syndrome Epilepsies, Myoclonic |
| 33 | Evrysdi | Risdiplam | Risdiplam | M09AX10 | Muscular Atrophy, Spinal |
| 34 | Farydak | Panobinostat | Panobinostat lactate anhydrous | L01XX42 | Multiple Myeloma |
| 35 | Fintepla | Fenfluramine | Fenfluramine hydrochloride | N03 | Epilepsies, Myoclonic |
| 36 | Galafold | Migalastat | Migalastat hydrochloride | A16AX | Fabry Disease |
| 37 | Gazyvaro | Obinutuzumab | Obinutuzumab | L01XC15 | Leukemia, Lymphocytic, Chronic, B-Cell |
| 38 | Givlaari | Givosiran | Givosiran | n/a | Porphyrias, Hepatic |
| 39 | Granupas | Para-aminosalicylic acid | Para-aminosalicylic acid | J04AA01 | Tuberculosis |
| 40 | Hepcludex | Bulevirtide | Bulevirtide acetate | J05A | Hepatitis D, Chronic |
| 41 | Hetlioz | Tasimelteon | Tasimelteon | N05CH | Sleep Disorders, Circadian Rhythm |
| 42 | Holoclar | Ex vivo expanded autologous human corneal epithelial cells containing stem cells | Ex vivo expanded autologous human corneal epithelial cells containing stem cells | S01XA19 | Stem Cell Transplantation Corneal Diseases |
| 43 | Iclusig | Ponatinib | Ponatinib | L01XE24 | Leukemia, Myeloid Leukemia, Lymphoid |
| 44 | Idefirix | Imlifidase | Imlifidase | L04AA | Desensitization, Immunologic; Kidney Transplantation |
| 45 | Idelvion | Albutrepenonacog alfa | Albutrepenonacog alfa | B02BD04 | Hemophilia B |
| 46 | Imbruvica | Ibrutinib | Ibrutinib | L01XE27 | Lymphoma, Mantle-Cell Leukemia, Lymphocytic, Chronic, B-Cell |
| 47 | Imcivree | Setmelanotide | Setmelanotide | A08AA | Obesity |
| 48 | Imnovid | Pomalidomide | Pomalidomide | L04AX06 | Multiple Myeloma |
| 49 | Inrebic | Fedratinib | fedratinib dihydrochloride monohydrate | L01EJ02 | Myeloproliferative Disorders; Primary Myelofibrosis |
| 50 | Insurisa | Osilodrostat | Osilodrostat phosphate | H02CA02 | Cushing Syndrome |
| 51 | Jorveza | Budesonide | Budesonide | A07EA06 | Esophageal Diseases |
| 52 | Kaftrio | Ivacaftor, tezacaftor, elexacaftor | Ivacaftor tezacaftor elexacaftor | R07AX32 | Cystic Fibrosis |
| 53 | Kalydeco | Ivacaftor | Ivacaftor (iwakaftor) | R07AX02 | Cystic Fibrosis |
| 54 | Kanuma | Sebelipase alfa | Sebelipase alfa | A16 | Lipid Metabolism, Inborn Errors |
| 55 | Ketoconazole HRA | Ketoconazole | Ketoconazole | J02AB02 | Cushing Syndrome |
| 56 | Kymriah | Tisagenlecleucel | Tisagenlecleucel | L01 | Precursor B-Cell Lymphoblastic Leukemia-Lymphoma Lymphoma, Large B-Cell, Diffuse |
| 57 | Kyprolis | Carfilzomib | Carfilzomib | L01XX45 | Multiple Myeloma |
| 58 | Lamzede | Velmanase alfa | Velmanase alfa | A16AB15 | alpha-Mannosidosis |
| 59 | Ledaga | Chlormethine | Chlormethine | L01AA05 | Mycosis Fungoides |
| 60 | Libmeldy | Autologous CD34+ cells encoding ARSA gene | atidarsagene autotemcel | N07 | Leukodystrophy, Metachromatic |
| 61 | Lutathera | Lutetium (177Lu) oxodotreotide | Lutetium (177Lu) oxodotreotide | V10XX04 | Neuroendocrine Tumors |
| 62 | Luxturna | Voretigene neparvovec | Voretigene neparvovec | n/a | Leber Congenital Amaurosis, Retinitis Pigmentosa |
| 63 | Mepsevii | Vestronidase alfa | Vestronidase alfa | A16AB18 | Mucopolysaccharidosis VII |
| 64 | Myalepta | Metreleptin | Metreleptin | A16AA | Lipodystrophy, Familial Partial |
| 65 | Mylotarg | Gemtuzumab ozogamicin | Gemtuzumab ozogamicin | L01XC05 | Leukemia, Myeloid, Acute |
| 66 | Namuscla | Mexiletine hcl | Mexiletine hcl | C01BB02 | Myotonic Disorders |
| 67 | Natpar | Parathyroid hormone | Parathyroid hormone | H05AA03 | Hypoparathyroidism |
| 68 | Nexavar | Sorafenib | Sorafenib | L01XE05 | Carcinoma, Hepatocellular Carcinoma, Renal Cell |
| 69 | NexoBrid | Concentrate of proteolytic enzymes enriched in bromelain | Concentrate of proteolytic enzymes enriched in bromelain | D03BA03 | Debridement |
| 70 | Ninlaro | Ixazomib | Ixazomib citrate | L01XX50 | Multiple Myeloma |
| 71 | Obiltoxaximab SFL | Obiltoxaximab | Obeticholic acid | A05AA04 | Anthrax |
| 72 | Ocaliva | Obeticholic acid | Nintedanib | L01XE | Liver Cirrhosis, Biliary |
| 73 | Onivyde pegylated liposomal | Irinotecan hydrochloride trihydrate | Irinotecan hydrochloride trihydrate | L01XX19 | Pancreatic Neoplasms |
| 74 | Onpattro | Patisiran | Patisiran sodium | N07 | Amyloidosis, Familial |
| 75 | Opsumit | Macitentan | Macitentan | C02KX04 | Hypertension, Pulmonary |
| 76 | Orphacol | Cholic acid | Cholic acid | A05AA03 | Digestive System Diseases Metabolism, Inborn Errors |
| 77 | Oxervate | Cenegermin | Recombinant human Nerve Growth factor (rhNGF) | S01 | Keratitis |
| 78 | Oxlumo | Lumasiran | Lumasiran sodium | A16AX18 | Hyperoxaluria, Primary |
| 79 | Palynziq | Pegvaliase | Pegvaliase | A16AB19 | Phenylketonurias |
| 80 | Pemazyre | Pemigatinib | pemigatinib | L01EX20 | Cholangiocarcinoma |
| 81 | Plenadren | Hydrocortisone | Hydrocortisone | H02AB09 | Adrenal Insufficiency |
| 82 | Polivy | Polatuzumab vedotin | Polatuzumab vedotin | L01XC | Lymphoma, B-Cell |
| 83 | Poteligeo | Mogamulizumab | Mogamulizumab | L01XC25 | Sezary Syndrome Mycosis Fungoides |
| 84 | Prevymis | Letermovir | Letermovir | J05 | Cytomegalovirus Infections |
| 85 | Procysbi | Mercaptamine | Mercaptamine bitartrate | A16AA04 | Cystinosis |
| 86 | Qarziba | Dinutuximab beta | Dinutuximab beta | L01XC | Neuroblastoma |
| 87 | Ravicti | Glycerol phenylbutyrate | Glycerol phenylbutyrate | A16AX09 | Urea Cycle Disorders, Inborn |
| 88 | Raxone | Idebenone | Idebenone | N06BX13 | Optic Atrophy, Hereditary, Leber |
| 89 | Reblozyl | Luspatercept | Luspatercept | B03XA06 | Anemia; Myelodysplastic Syndromes; beta-Thalassemia |
| 90 | Revestive | Teduglutide | Teduglutide | A16AX08 | Malabsorption Syndromes |
| 91 | Rydapt | Midostaurin | Midostaurin | L01XE | Leukemia, Myeloid, Acute Mastocytosis |
| 92 | Scenesse | Afamelanotide | Afamelanotide | D02BB02 | Protoporphyria, Erythropoietic |
| 93 | Signifor | Pasireotide | Pasireotide | H01CB05 | Acromegaly Pituitary ACTH Hypersecretion |
| 94 | Sirturo | Bedaquiline | Bedaquiline fumarate | J04AK05 | Tuberculosis, Multidrug-Resistant |
| 95 | Skysona | Elivaldogene autotemcel | elivaldogene autotemcel | N07 | Adrenoleukodystrophy |
| 96 | Sogroya | Somapacitan | Somapacitan | n/a | Growth |
| 97 | Soliris | Eculizumab | Eculizumab | L04AA25 | Hemoglobinuria, Paroxysmal |
| 98 | SomaKit TOC | Edotreotide | Edotreotide | V09IX | Neuroendocrine Tumors Radionuclide Imaging |
| 99 | Spinraza | Nusinersen | Nusinersen sodium | M09 | Muscular Atrophy, Spinal |
| 100 | Strensiq | Asfotase alfa | Asfotase alfa | A16AB | Hypophosphatasia |
| 101 | Strimvelis | Autologous CD34+ enriched cell fraction that contains CD34+ cells transduced with retroviral vector that encodes for the human ADA cDNA sequence | Autologous CD34+ enriched cell fraction that contains CD34+ cells transduced with retroviral vector that encodes for the human adenosine deaminase (ADA) cDNA sequence from human haematopoietic stem/progenitor (CD34+) cells | L03 | Severe Combined Immunodeficiency |
| 102 | Sylvant | Siltuximab | Siltuximab | L04AC11 | Giant Lymph Node Hyperplasia |
| 103 | Symkevi | Tezacaftor, Ivacaftor | Tezacaftor Ivacaftor | R07AX31 | Cystic Fibrosis |
| 104 | Takhzyro | Lanadelumab | Lanadelumab | B06AC05 | Angioedemas, Hereditary |
| 105 | Tecartus | Autologous peripheral blood T cells CD4 and CD8 selected and CD3 and CD28 activated transduced with retroviral vector expressing anti-CD19 CD28/CD3-zeta chimeric antigen receptor and cultured | Autologous peripheral blood T cells CD4 and CD8 selected and CD3 and CD28 activated transduced with retroviral vector expressing anti-CD19 CD28/CD3-zeta chimeric antigen receptor and cultured | L01X | Lymphoma, Mantle-Cell |
| 106 | Tegsedi | Inotersen | Inotersen sodium | N07 | Amyloidosis |
| 107 | Tobi Podhaler | Tobramycin | Tobramycin | J01GB01 | Cystic Fibrosis Respiratory Tract Infections |
| 108 | Translarna | Ataluren | Ataluren | M09AX03 | Muscular Dystrophy, Duchenne |
| 109 | Trecondi | Treosulfan | Treosulfan | L01AB02 | Hematopoietic Stem Cell Transplantation |
| 110 | Trepulmix | Treprostinil | Treprostinil sodium | B01AC21 | Hypertension, Pulmonary |
| 111 | Verkazia | Ciclosporin | Ciclosporin | S01XA18 | Conjunctivitis Keratitis |
| 112 | Vimizim | Elosulfase alfa | Recombinant human n-acetylgalactosamine-6-sulfatase (rhgalns) | A16AB12 | Mucopolysaccharidosis IV |
| 113 | Votubia | Everolimus | Everolimus | L01XE10 | Tuberous Sclerosis |
| 114 | Vpriv | Velaglucerase alfa | Velaglucerase alfa | A16AB10 | Gaucher Disease |
| 115 | Vyndaqel | Tafamidis | Tafamidis | N07XX08 | Amyloidosis |
| 116 | Vyxeos liposomal | Daunorubicin, cytarabine | Daunorubicin hydrochloride / cytarabine | L01XY01 | Leukemia, Myeloid, Acute |
| 117 | Wakix | Pitolisant | Pitolisant | N07XX11 | Narcolepsy |
| 118 | Waylivra | Volanesorsen | Volanesorsen sodium | n/a | Hyperlipoproteinemia Type I |
| 119 | Xaluprine | mercaptopurine | 6-mercaptopurine monohydrate | L01BB02 | Leukemia, Lymphoid |
| 120 | Xermelo | telotristat ethyl | Telotristat etiprate | A16A | Carcinoid Tumor Neuroendocrine Tumors |
| 121 | Xospata | Gilteritinib | Gilteritinib fumarate | L01XE54 | Leukemia, Myeloid, Acute |
| 122 | Yescarta | Axicabtagene ciloleucel | Axicabtagene ciloleucel | L01X | Lymphoma, Follicular Lymphoma, Large B-Cell, Diffuse |
| 123 | Zejula | Niraparib | Niraparib (tosylate monohydrate) | L01XX | Fallopian Tube Neoplasms Peritoneal Neoplasms Ovarian Neoplasms |
| 124 | Zolgensma | Onasemnogene abeparvovec | onasemnogene abeparvovec | M09AX09 | Muscular Atrophy, Spinal |
| 125 | Zynteglo | Betibeglogene autotemcel | Autologous CD34+ cell enriched population that contains hematopoietic stem cells transduced with lentiglobin BB305 lentiviral vector encoding the beta-A-T87Q-globin gene | B06A | Beta-Thalassemia |

Source: European Medicines Agency. Medicines. https://www.ema.europa.eu/en/medicines (accessed September 1, 2021).

# Appendix 2. | The concordance between positive recommendation and reimbursement by country, drug type, and ATC classification.

|  |  | **Bulgaria** | **Croatia** | **Czechia** | **Estonia** | **Hungary** | **Poland** | **Romania** | **Slovakia** | **All** |
| --- | --- | --- | --- | --- | --- | --- | --- | --- | --- | --- |
| All drugs | Agreement | 100% | 100% | 78.8% | 90.3% | 64.0% | 80.8% | 89.4% | 80.8% | 85.5% |
|  | Cohen’s κ | - | - | 0.573 | 0.578 | 0.276 | 0.448 | 0.778 | 0.392 | 0.644 |
|  | p-value | - | - | <0.001 | <0.001 | <0.001 | <0.001 | <0.001 | <0.001 | <0.001 |
| ATC A | Agreement | 100% | 100% | 78.3% | 91.7% | 54.2% | 87.5% | 91.7% | 83.3% | 85.9% |
|  | Cohen’s κ | - | - | 0.531 | n/a | n/a | 0.600 | 0.800 | 0.429 | 0.589 |
|  | p-value | - | - | 0.002 | n/a | n/a | 0.001 | <0.001 | 0.005 | <0.001 |
| ATC B | Agreement | 100% | 100% | 77.8% | 80.0% | 90.0% | 70.0% | 77.8% | 60.0% | 82.1% |
|  | Cohen’s κ | - | - | 0.526 | n/a | 0.737 | n/a | 0.400 | n/a | 0.463 |
|  | p-value | - | - | 0.037 | n/a | 0.008 | n/a | 0.067 | n/a | <0.001 |
| ATC J | Agreement | 100% | 100% | 80.0% | 100% | 60.0% | 90.0% | 100% | 80.0% | 88.8% |
|  | Cohen’s κ | - | - | 0.546 | - | 0.200 | n/a | - | 0.375 | 0.715 |
|  | p-value | - | - | 0.026 | - | 0.146 | n/a | - | 0.118 | <0.001 |
| ATC L | Agreement | 100% | 100% | 82.9% | 83.7% | 72.7% | 72.7% | 86.1% | 84.1% | 85.3% |
|  | Cohen’s κ | - | - | 0.667 | 0.507 | 0.477 | 0.411 | 0.723 | 0.547 | 0.692 |
|  | p-value | - | - | <0.001 | <0.001 | 0.001 | 0.003 | <0.001 | <0.001 | <0.001 |
| ATC N | Agreement | 100% | 100% | 88.9% | 100% | 50.0% | 90.0% | 90.0% | n/a | 89.9% |
|  | Cohen’s κ | - | - | 0.769 | - | n/a | n/a | 0.782 | n/a | 0.654 |
|  | p-value | - | - | 0.009 | - | n/a | n/a | 0.006 | n/a | <0.001 |
| other ATCs* | Agreement | 100% | 100% | 69.2% | 96.3% | 55.6% | 85.2% | 92.6% | 74.1% | 84.1% |
|  | Cohen’s κ | - | - | 0.302 | 0.781 | 0.085 | 0.509 | 0.809 | 0.276 | 0.558 |
|  | p-value | - | - | 0.016 | <0.001 | 0.248 | 0.004 | <0.001 | 0.019 | <0.001 |

ATC Anatomical Therapeutic Chemical | n/a not applicable due to partial separation
! Data from Lithuania were not obtained.
* other ATCs: M, H, R, S, C, D, V
